# Factors associated with the use of long-lasting insecticide-treated mosquito nets among women of childbearing age: secondary analysis of data from a KAP survey in four regions of Guinea 2023

**DOI:** 10.3389/fpubh.2026.1770818

**Published:** 2026-04-08

**Authors:** Thierno Ibrahima Diallo, Almamy Amara Toure, Houssainatou Bah, Abdoulaye Fodé Toure, Amadou Wann, Mohamed Prince Kaba

**Affiliations:** 1 Department of Public Health Research, National Institute of Public Health, Coyah, Guinea; 2 Department of Public Health, Faculty of Health Sciences and Technology, Gamal Abdel Nasser University, Conakry, Guinea

**Keywords:** attitudes, Guinea, KAP, knowledge, malaria, practice, women of childbearing age

## Abstract

**Background:**

Malaria is endemic in Guinea. The use of mosquito nets by women of childbearing age helps protect children under five from malaria.

**Aim:**

To estimate the proportion of mosquito nets use and identify the factors explaining that use among women aged 15 to 49 in four regions of Guinea.

**Setting:**

The study was carried out in Guinea specifically in Conakry, Boke, Kindia, and Labe regions.

**Methods:**

We conducted a secondary analysis of a 2023 cross-sectional study, yielding a sample of 1,876 women. The outcome was mosquito net use the night before the survey. Simple logistic regressions assessed associations with independent variables were included in multivariate analysis.

**Results:**

In our study, 64.23% of women slept under a mosquito net the night before the survey. Living in an urban area is associated with a reduced likelihood of sleeping under a mosquito net (OR = 0.54; 95% CI = 0.43–0.68). Believing that sleeping under a mosquito net poses no health risk significantly increases the likelihood of using it (OR = 2.14; 95% CI = 1.64–2.79), as does the belief that mosquito nets are very useful (OR = 2.33; 95% CI = 1.38–4.00).

**Conclusion:**

These results suggest that communication strategies must focus on transforming women’s knowledge and attitudes by using approaches that are more engaging, participatory, and adapted to local realities.

**Contribution:**

This study shows that knowledge and attitudes toward malaria matter when it comes to preventive practices like the use of mosquito nets among women in childbearing age.

## 1 Introduction

Malaria is a serious health problem in Africa (1). In 2023, nearly 246 million cases of malaria were recorded in the World Health Organization (WHO) African Region, which continues to bear the heaviest burden of malaria (2). The use of long-lasting insecticide-treated mosquito nets (LLINs) is one of the most effective malaria prevention strategies (3). Universal coverage of populations with LLINs is a prevention strategy recommended by the WHO in malaria control programs (4). Their use has been shown to reduce the incidence of malaria episodes by 50% in endemic areas and has therefore become one of the key strategies employed in the global response to malaria (5). Furthermore, between 2000 and 2019, 67% of the reduction in malaria mortality was attributed to the intensification of LLINs use and behavior change campaigns, which encouraged their use (6). However, ownership does not necessarily guarantee their use, and the greatest challenges relate to their effective use by individuals and the replacement of old and torn mosquito nets (1). Indeed, even in the ideal context where universal coverage by LLINS has been achieved, maximum protection against malaria will only be obtained if they are used correctly and systematically (5).

Malaria is endemic in Guinea and is the leading cause of clinical consultations, hospitalizations, and deaths in hospitals in the country (7). It is the leading public health problem, accounting for nearly 47.5% of hospital morbidity (8) with more than 4 million cases and 10,000 deaths in 2021 among a population of approximately 13 million (9). The heaviest burden of this disease is bore by populations that have not yet acquired complete immunity to malaria, such as children under 5 years of age, or those whose immunity has been suppressed, for example by physiological conditions such as pregnancy in women (10).

Women of childbearing age (15–49 years) can become pregnant at any time and are part of the population vulnerable to malaria. They are also generally more likely to share their sleeping space with children under 5 years of age; therefore, their mosquito net usage habits help protect children under five from malaria (11). That is why efforts must be stepped up, particularly in raising awareness among the population for greater use of LLINs (12).

In 2016, 54% of women aged 15 to 49 slept under LLINs on the night before the multiple indicator cluster survey (13). In 2018, only 44% of households had mosquito nets, while in 2021, this proportion rose to 63%, indicating a significant increase in prevention coverage (14). According to the 2021 Guinea Malaria and Anemia Indicator Survey, 42% of the household population had access to an LLIN and 33% reported sleeping under an LLIN the night before the interview, resulting in a 9 percentage point gap between access to and use of LLINs (12).

Studies in Guinea focus on malaria epidemiology, drug efficacy, and resistance, while behavioral and social factors such as mosquito net use remain largely understudied (15).

Few studies have explored the factors associated with the use of LLINs among women in childbearing age in Guinea. Thus, the objective of this study was to estimate the proportion of LLINs use and identify the factors explaining their use among women aged 15 to 49 in four regions of Guinea based on data from the KAP survey.

*Hypothesis*: We hypothesize that knowledge about malaria and attitudes toward LLINs influence the strength and significance of associations between exposure variables (type of residence, age, marital status, number of children under five, pregnancy) and LLIN use on the night before the survey.

## 2 Materials and methods

### 2.1 Study design

This was a secondary analysis based on data from a cross-sectional observational study conducted in 2023 in four regions of Guinea: Conakry, Boke, Labe, and Kindia.

### 2.2 Setting

**Figure 1:**
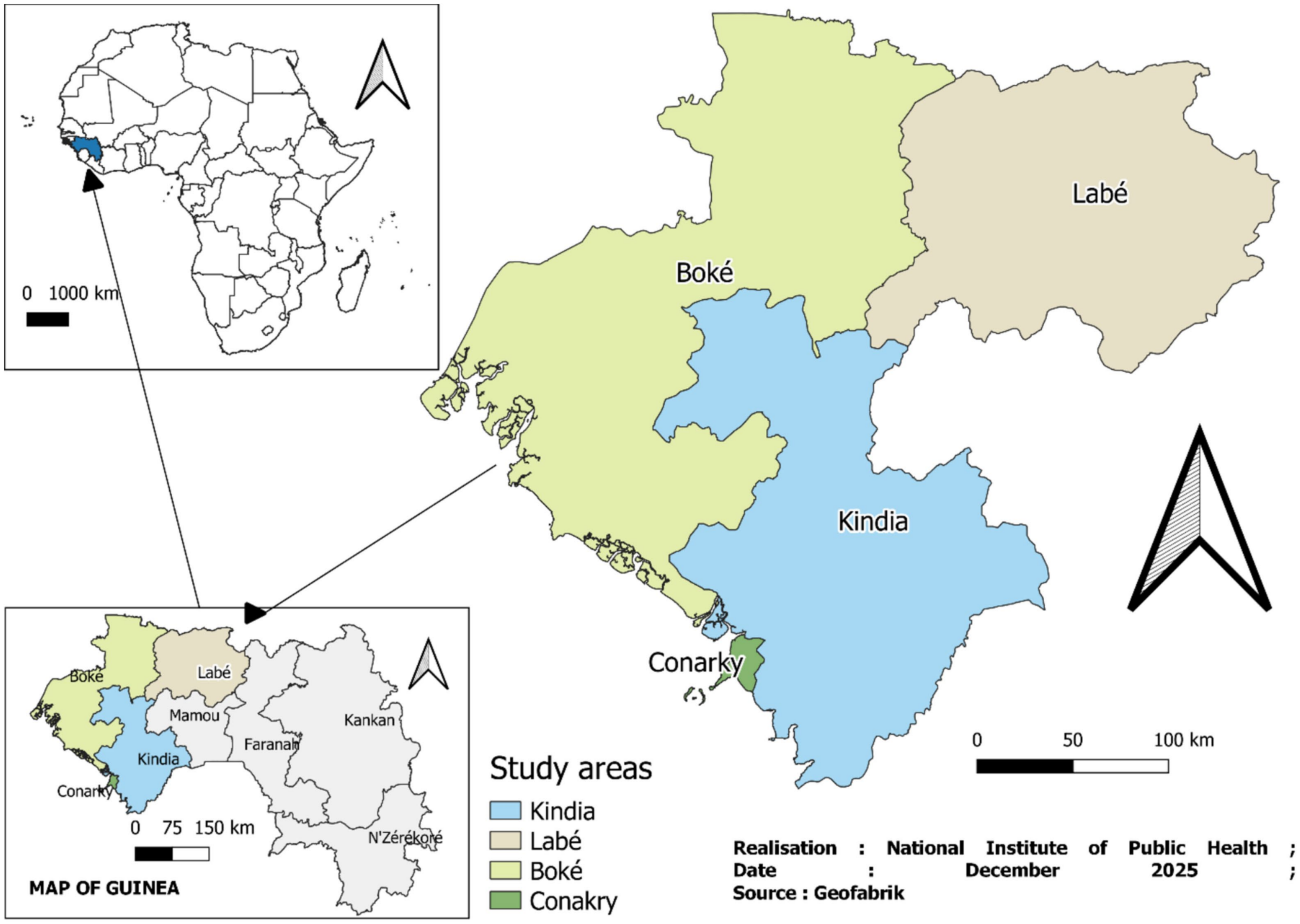
Map of study areas included in the 2023 KAP survey, Guinea.

The research activities were carried out the 4 regions of Labe, Kindia, Boke, and Conakry. Figure 1 shows the regions where the study was carried out.

### 2.3 Study population and sampling strategy

**Figure 2:**
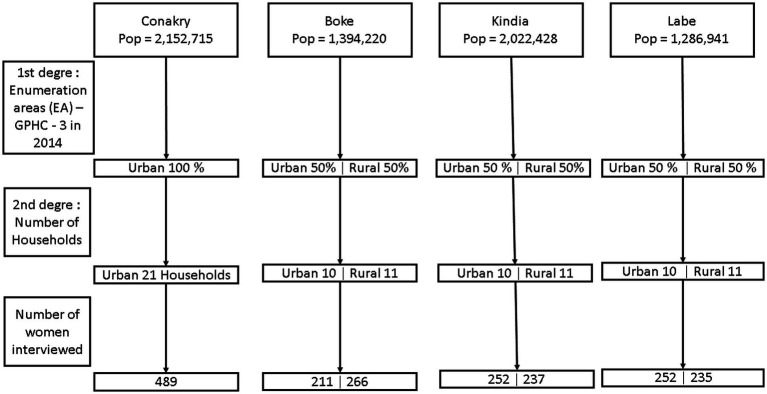
Sampling distribution.

The original study used two-stage sampling. In the first stage, primary units or enumeration areas (EA) were selected with a probability proportional to their size at the stratum level. Thus, 50% of EAs were selected in rural areas in the regions of Kindia, Boke, and Labe. In Conakry, all EAs were urban. The EAs were drawn proportionally to the weight of each prefecture in the region, both in rural and urban areas. In the second stage, all eligible households were selected. As shown in Figure 2, the sampling design was stratified by region with separate subsamples drawn from rural and urban areas. The figure presents the total population of each region alongside the corresponding sample size. The route method was used in this household survey. It consisted of establishing predefined routes that the interviewers followed to collect data from households. The instruction was to interview every other household (14).

#### 2.3.1 Study population—KAP survey study period

The KAP survey was conducted between August 16 and December 16, 2023. The study population consisted of women who met the inclusion/exclusion criteria listed below.

##### 2.3.1.1 Inclusion criteria

- Women of childbearing age (15 to 49 years old): and who have given birth to a child in the last 5 years.
- Give their free and informed consent to participate in the survey.

##### 2.3.1.2 Exclusion criteria

Participants who did not agree to take part in the survey were not included in the study.

#### 2.3.2 Population of our study

Our study population consisted of women with complete data on sociodemographic variables, knowledge and attitude variables.

**Figure 3:**
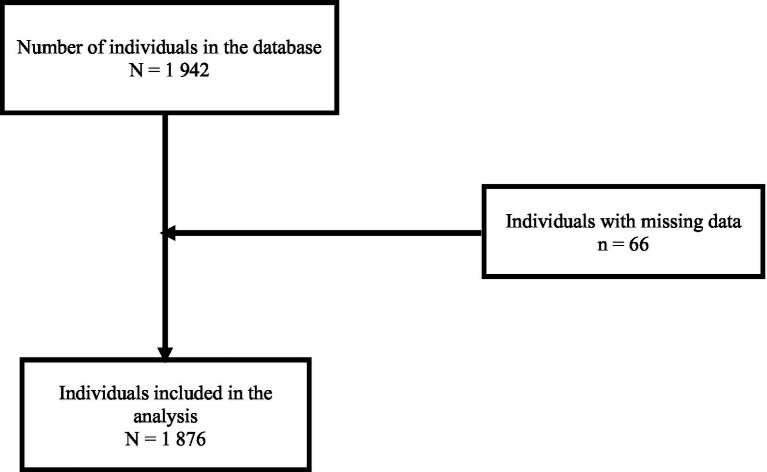
Study flow chart of participant selection and inclusion in the study.

After receiving the database in Excel format, we imported into R Studio 4.4.0 for all analyses. The initial dataset included 1,942 individuals. Individuals with missing data on knowledge, attitudes, and practices variables were excluded (*n* = 66) to ensure the consistency of the statistical models and the validity of the estimates. Therefore, the final sample size comprised 1,876 individuals. Figure 3 gives details about individuals inclusion.

### 2.4 Data collection and variables used

*Knowledge*: the body of information known, understood, and acquired about a given subject. This includes informational data or facts that a person has assimilated concerning a specific field (16).

*Attitudes*: refers to a posture, a positioning, a predisposition to act in a particular way (favourable or unfavourable) with regard to a person, a group, an idea (16).

In this study, the data used are secondary, they were not collected directly from participants but obtained from existing source. Using secondary data provides access to reliable and validated information while avoiding the constraints of primary data collection such as surveys or direct observations. However, it is important to note that the quality and relevance of the findings depend on the rigor of the sources consulted and the methods by which the data were originally produced. The data collection method was direct interviews with women. Data were collected using tablets via mobile platforms. In order to frame our study, we selected the variables deemed relevant to our analytical objective. Thus, after reviewing the literature on factors associated with the use of LLINs among women, we selected the variables detailed in Table 1.

**Table 1:** Description of study variables and measurement modalities.

No	Variables	Modalities
I-Dependent variable		
1	Did you sleep under an LLIN last night?	Yes, no
II-Exposure variables (socioeconomic)		
2	Type of residential environment	Urban, rural
3	Age of the woman	<30 years old and ≥30 years old
4	Marital status	Single, married, divorced, and widowed
5	Education of the woman	None, Primary, Secondary, and Higher
6	Number of children under 5 living in the household	1 child, 2 children, and more than 2 children
7	Pregnant woman	Yes, no
III-Adjustment variables		
III-1 Knowledge of malaria		
8	Is malaria caused by mosquito bites?	True and false
9	Does sleeping under an LLIN prevent malaria?	True and false
III-2 General attitudes toward malaria and LLINs		
10	Sleeping under an insecticide-treated mosquito net poses no risk to my health.	Agree, do not know/not sure, and disagree
11	Mosquito nets are very useful.	Agree, do not know/not sure, and disagree
12	The likelihood of me getting malaria is the same whether I sleep under a mosquito net or not.	Agree, do not know/not sure, and disagree
13	Sleeping under a mosquito net every night is the best way to avoid malaria.	Agree, do not know/not sure, and disagree
14	Many people who sleep under mosquito nets still get malaria.	Agree, do not know/not sure, and disagree

### 2.5 Data analysis

The descriptive analysis consisted of describing the modalities of the socioeconomic variables according to their numbers and associated percentages. The quantitative variables were all transformed into qualitative variables, as explained in the exposure variables. All variables were therefore presented with their numbers and percentages. At this level, we performed simple logistic regressions to explore the association between each independent variable and the dependent variable. Crude ORs, 95% confidence intervals, and *p*-values were calculated. Variables with a *p*-value <0.20 were retained for multivariate analysis.

Multivariate analysis was conducted using logistic regression to identify factors associated with the use of LLINs by women of childbearing age on the night before the survey. Two models were constructed to assess the effect of adjustment variables on the observed associations:

- Model 1: includes only exposure variables (socioeconomic variables).
- Model 2: includes the same exposure variables with the addition of adjustment variables.

The objective of this dual modelling is to test the hypothesis that adjustment variables influence the strength and significance of associations between women’s individual characteristics and their use of LLINs.

A test of the simultaneous contribution of a subset of explanatory variables (likelihood ratio test or log-likelihood test) was used to assess whether the addition of adjustment variables significantly improves the quality of the model. Statistical significance was set at *p* < 0.05.

Before selecting the variables useful for the model, a collinearity test will be performed on model 2 if it is statistically different from model 1 by searching for generalized variance inflation factors (VIF). A VIF greater than 5 will be a sign of multicollinearity of a variable with one or more other independent variables in the model.

To optimize the model structure while avoiding overfitting, automatic variable selection was performed using the stepwise selection method based on the Akaike Information Criterion (AIC) criterion. This approach identified a more parsimonious final model, retaining the most informative variables and avoiding overfitting. The results are presented as adjusted odds ratios (ORa), accompanied by their 95% confidence intervals. The goodness of fit of the final model was assessed using the Hosmer-Lemeshow test. This test examines the agreement between predicted probabilities and actual observations. A non-significant result (*p* > 0.05) indicates that the model fits the data well.

### 2.6 Ethical considerations

The initial study protocol was submitted in advance to the National Ethics Committee for Health Research (CNERS) for evaluation and approval. This approval was obtained under number L-175/CNERS/23. Before the study began, all participants provided their free and informed consent during a process carried out in the presence of only the investigator and the participant. In order to guarantee the confidentiality of the information shared, the data used was anonymized (14). Their use was authorized, first by the principal investigator and then by the Department of Health and Human Services.

## 3 Results

### 3.1 Description of the population of women surveyed

In this study 1,876 women were involved. The urban environment was the most represented, with 62.05% of women residing in this environment. More than half of the women had received no formal education (53.68%), followed by primary education (20.26%). The age group of women under 30 was the largest, accounting for 62.10% of cases. The average age was 27.40 years with a standard deviation of 6.61 years. Only 11.89% of those surveyed were pregnant at the time of the survey. Table 2 shows the results of the description of the socioeconomic characteristics of the women surveyed.

**Table 2:** Socioeconomic characteristics of the 1,876 women of childbearing age from four regions of Guinea (KAP survey, 2023).

Socioeconomic characteristics	*N* = 1876 (%)
Type of residential environment	
Rural	712 (37.95)
Urban	1164 (62.05)
Highest level of education attained	
No formal education	1007 (53.68)
Vocational school	81 (4.32)
Elementary school	380 (20.26)
High school	317 (16.90)
University	91 (4.85)
Marital status	
Single	77 (4.10)
Divorced	13 (0.69)
Married	1768 (94.24)
Widowed	18 (0.96)
Age group	
Under 30	1165 (62.10)
30 or older	711 (37.90)
Classification based on the number of children under 5 in the household	
1 child	1118 (59.59)
2 children	625 (33.32)
More than 2 children	133 (7.09)
Are you currently pregnant?	
No	1653 (88.11)
Yes	223 (11.89)

Average age = 27.41 years; Standard deviation = 6.61 years.

### 3.2 Description according to LLINS usage

In the study population, 1,205 women slept under a mosquito net the night before the survey, representing a relative frequency of 64.23% [95% CI: (62.1–66.4%)] compared to 671 who did not sleep under a LLINS, representing a relative frequency of 35.77% [95% CI: (33.6–37.9%)].

### 3.3 Univariate and multivariate regressions

The results of the analyses are summarized in Table 3. The univariate regression shows that all variables are associated with the use of LLINs by women, except for the variable “Number of children under 5 in the household,” which has a *p*-value of 0.8 and will therefore not be included in the models for the rest of the analysis. The multivariate regression was divided into two models, which were compared.

**Table 3:** Univariate regression results and multivariate models assessing LLIN use among women of childbearing age (KAP survey, 2023).

Features	*N*	Univariate model			Multivariate model: exposure			Multivariate model: exposure + adjustment		
		OR^a^	95% IC^a^	*p*-value	ORa	95% IC^a^	*p*-value	ORa	95% IC^a^	*p*-value
Type of residential environment	1876			**<0.001**			**<0.001**			**<0.001**
Rural		—	—		—	—		—	—	
Urban		0.50	0.41–0.61	**<0.001**	0.87	0.83–0.91	**<0.001**	0.89	0.85–0.93	**<0.001**
Highest level of education attained	1876			**<0.001**			**<0.001**			**<0.001**
No formal education		—	—		—	—		—	—	
Vocational school		1.30	0.79–2.22	0.3	1.12	1.00–1.25	**0.041**	1.06	0.96–1.18	0.3
Elementary school		0.65	0.51–0.83	**<0.001**	0.93	0.88–0.99	**0.018**	0.92	0.87–0.97	**0.003**
High school		0.51	0.40–0.66	**<0.001**	0.91	0.85–0.97	**0.003**	0.90	0.85–0.96	**<0.001**
University		0.93	0.59–1.48	0.7	1.05	0.95–1.17	0.3	1.02	0.92–1.12	0.7
Marital status	1876			**0.003**			**0.048**			0.2
Single		—	—		—	—		—	—	
Divorced		2.02	0.62–7.23	0.3	1.19	0.91–1.57	0.2	1.07	0.82–1.39	0.6
Married		2.37	1.50–3.77	**<0.001**	1.17	1.05–1.30	**0.005**	1.12	1.01–1.24	**0.032**
Widowed		1.99	0.71–5.92	0.2	1.12	0.88–1.43	0.3	1.14	0.90–1.43	0.3
Age groups	1876			0.068			0.12			0.3
Under 30		—	—		—	—		—	—	
30 or older		1.20	0.99–1.46	0.069	1.04	0.99–1.08	0.12	1.02	0.98–1.07	0.3
Classification based on the number of children under 5 in the household	1876			0.8						
1 child		—	—							
2 children		1.05	0.86–1.29	0.6						
More than 2 children		0.94	0.65–1.36	0.7						
Pregnancy	1876			**0.038**			0.10			**0.035**
No		—	—		—	—		—	—	
Yes		1.37	1.02–1.87	**0.041**	1.06	0.99–1.13	0.10	1.07	1.00–1.14	**0.035**
Is malaria caused by mosquito bites?	1876			**0.005**						0.078
False		—	—					—	—	
True		1.65	1.17–2.32	**0.004**				1.07	0.99–1.16	0.078
Does sleeping under a LLIN prevent malaria?	1876			**0.001**						0.2
False		—	—					—	—	
True		1.36	1.12–1.64	**0.001**				1.03	0.98–1.07	0.2
Sleeping under an insecticide-treated mosquito net poses no risk to my health.	1876			**<0.001**						**<0.001**
Disagree		—	—					—	—	
Agree		2.15	1.69–2.74	**<0.001**				1.17	1.11–1.24	**<0.001**
Do not know/not sure		0.35	0.22–0.54	**<0.001**				0.91	0.83–1.01	0.089
Mosquito nets are very useful	1876			**<0.001**						**<0.001**
Disagree		—	—					—	—	
Agree		3.42	2.12–5.62	**<0.001**				1.20	1.07–1.33	**0.001**
Do not know/not sure		0.53	0.24–1.13	0.10				1.03	0.87–1.22	0.7
The probability of me getting malaria is the same. Whether I sleep under a mosquito net or not	1876			**<0.001**						0.12
Disagree		—	—					—	—	
Agree		0.95	0.78–1.16	0.6				1.03	0.98–1.08	0.2
Do not know/not sure		0.32	0.22–0.47	**<0.001**				0.94	0.85–1.03	0.2
Sleeping under a mosquito net every night is the best way to avoid malaria	1876			**<0.001**						**<0.001**
Disagree		—	—					—	—	
Agree		3.35	2.44–4.63	**<0.001**				1.19	1.10–1.28	**<0.001**
Do not know/not sure		0.52	0.26–0.98	**0.049**				1.05	0.90–1.22	0.5
Many people who sleep under mosquito nets still get malaria	1876			**<0.001**						**<0.001**
Disagree		—	—					—	—	
Agree		0.63	0.51–0.77	**<0.001**				0.91	0.87–0.95	**<0.001**
Do not know/not sure		0.32	0.22–0.46	**<0.001**				0.90	0.82–0.98	**0.017**

a OR, Odds ratio; IC, confidence interval.Bold values are significative *p*-values.

There was a significant difference between Model 1, which included only exposure variables, and Model 2, which included both exposure and adjustment variables (*p* = 2.2*e*−16). This indicates that incorporating malaria-related knowledge and attitudes improves the explanation of LLIN use. In Model 1, pregnancy was not significantly associated with LLIN use; however, in Model 2, pregnancy was associated with a higher probability of LLIN use after adjustment for other variables (OR = 1.07; 95% CI: 1.00–1.14). After adjustment in Model 2, all variables were significantly associated with LLIN use except marital status (*p* = 0.20), age groups (*p* = 0.30), and the following specific knowledge and attitude items: “Is malaria caused by mosquito bites?” (*p* = 0.078), “Does sleeping under an LLIN prevent malaria?” (*p* = 0.20), and “The probability of me getting malaria is the same whether I sleep under a mosquito net or not” (*p* = 0.12). Overall, the selected model provided a good compromise between parsimony and performance (Table 4).

**Table 4:** Variables retained through stepwise logistic regression using the AIC.

Features	OR^a^	95% IC^a^	*p*-value
Type of residential environment			<0.001
Rural	—	—	
Urban	0.54	0.43–0.68	<0.001
Highest level of education attained			<0.001
No formal education	—	—	
Vocational school	1.37	0.80–2.43	0.3
Elementary school	0.66	0.50–0.86	0.002
High school	0.60	0.45–0.80	<0.001
University	1.09	0.67–1.82	0.7
Pregnancy			0.023
No	—	—	
Yes	1.46	1.05–2.05	0.026
Is malaria caused by mosquito bites?			0.049
False	—	—	
True	1.48	1.00–2.18	0.048
Sleeping under an insecticide-treated mosquito net poses no risk to my health			<0.001
Disagree	—	—	
Agree	2.14	1.64–2.79	<0.001
Do not know/not sure	0.66	0.39–1.08	0.10
Mosquito nets are very useful			<0.001
Disagree	—	—	
Agree	2.33	1.38–4.00	0.002
Do not know/not sure	0.95	0.38–2.32	>0.9
The probability of me getting malaria is the same. Whether I sleep under a mosquito net or not			0.10
Disagree	—	—	
Agree	1.18	0.93–1.50	0.2
Do not know/not sure	0.73	0.46–1.16	0.2
Sleeping under a mosquito net every night is the best way to avoid malaria			<0.001
Disagree	—	—	
Agree	2.27	1.60–3.23	<0.001
Do not know/not sure	1.26	0.56–2.77	0.6
Many people who sleep under mosquito nets still get malaria			<0.001
Disagree	—	—	
Agree	0.61	0.48–0.78	<0.001
Do not know/not sure	0.56	0.36–0.86	0.008

Hosmer-Lemeshow test: Chi^2^ = 12.43, ddl = 8, *p* = 0.1329.

a OR, Odds ratio; IC, confidence interval.

See the model diagnostics (collinearity, AIC-based selection, and calibration) in the Supplementary material.

The final model highlights several factors significantly associated with the outcome studied. The type of residential environment appears to be a major determinant: living in an urban area is associated with a reduced likelihood of sleeping under LLINs (OR = 0.54; 95% CI = 0.43–0.68; *p* < 0.001), compared to rural areas. Level of education also plays a role: compared to those with no formal education, individuals who have completed primary school (OR = 0.66; 95% CI = 0.50–0.86; *p* = 0.002) or secondary school (OR = 0.60; 95% CI = 0.45–0.80; *p* < 0.001) are significantly less likely to use LLINs, while university (OR = 1.09; 95% CI = 0.67–1.82; *p* = 0.7) and professional (OR = 1.37; 95% CI = 0.80–2.43; *p* = 0.3) did not show a significant effect. Pregnancy was associated with an increased likelihood of using LLINS (OR = 1.46; 95% CI = 1.05–2.05; *p* = 0.026).

Perceptions and beliefs about malaria strongly influence the use of LLINs by women of childbearing age. Believing that malaria is caused by mosquito bites is associated with a higher probability of adopting protective behaviors (OR = 1.48; 95% CI = 1.00–2.18; *p* = 0.048). Believing that sleeping under an insecticide-treated mosquito net poses no health risk significantly increases the likelihood of using LLINs (OR = 2.14; 95% CI = 1.64–2.79; *p* < 0.001), as does the belief that mosquito nets are very useful (OR = 2.33; 95% CI = 1.38–4.00; *p* = 0.002) or that they are the best way to avoid malaria (OR = 2.27; 95% CI = 1.60–3.23; *p* < 0.001). Conversely, believing that malaria can be contracted even when sleeping under a mosquito net is associated with reduced adherence (OR = 0.61; 95% CI = 0.48–0.78; *p* < 0.001), as is uncertainty expressed by respondents (OR = 0.56; 95% CI = 0.36–0.86; *p* = 0.008). These results highlight the importance of beliefs in the adoption of preventive measures.

## 4 Discussion

This study explored LLIN use among women in childbearing age. The discussion highlights the main findings, interprets them in relation to existing literature and considers their implications for malaria prevention strategies, with particular focus on knowledge and attitudes as key behavioral determinants of LLIN use. Knowledge was assessed through factual questions such as awareness that malaria is caused by mosquito bites, while attitudes were measured through perceptions and beliefs regarding malaria risk and the effectiveness of LLINs.

### 4.1 Description of the population of women surveyed

The urban environment was the most represented (62.05%). This result reflects the fact that Conakry (the capital of Guinea), the most populous city, is exclusively urban. However, this imbalance may limit the generalizability of LLIN use estimates. Urban participants often have different access to services and campaigns compared to rural population, so finding should be interpreted with caution.

More than half of the women had received no formal education (53.68%), followed by primary education (20.26%). A possible explanation for this finding is the generally low literacy rate in Guinea, particularly among young girls (17). Education is a well-known determinant of health behavior, influencing both access to information and the adoption of preventive practices. This underscores the need for tailored communication strategies that address population with low literacy.

### 4.2 Use of LLINs

During the survey, 64.23% of women slept under a LLIN the night before. This indicates relatively high use of LLINs, though more than one-third of women remain unprotected. This result differs from that in Chad, where 37.6% of respondents used LLINs the night before the survey (18). These results are consistent with those in Guinea that reported that 61.2% of respondents had slept under LLINs the night before the Multiple Indicator Cluster Survey 2016 (19). Strengthening behavioural adoption is essential to maximize malaria prevention among women in childbearing age.

### 4.3 Significant variables on use of LLINs

- *Type of residential environment*: women living in urban areas are less likely to sleep under an LLIN than women living in rural areas (OR = 0.54; 95% CI = 0.43–0.68), when the effect is adjusted for other variables in the model. Urban households may perceive lower malaria risk, housing structures that complicate net use or prioritize other preventive measures. In contrast, rural areas often experience higher exposure to mosquitoes and stronger reliance on LLINs. This result is consistent with that in Guinea, where the place of residence influenced compliance with malaria prevention rules among pregnant women (20). This result shows association that may inform targeted health promotion strategies in urban areas in order to change their behavior. Therefore, interventions should emphasize risk communication in urban areas in order to increase the adoption of LLIN as a preventive measure.
- *Marital status*: The effect of the variable on the use of LLINs is no longer significant when adjusted for knowledge and attitudes toward malaria, with a *p*-value = 0.2, and the variable no longer appears in the final model. This suggests that its apparent effect was largely explained by differences in knowledge and attitudes, rather than the variable itself. Knowledge and attitudes may act as stronger determinants, overshadowing the independent contribution of the variable. This result differs from findings reported in Sierra Leone, where married pregnant women were more likely to use a mosquito net than those who were not married (21). This difference suggests that in our study population, behavioral factors may play a more central role than marital status in influencing preventive practices. Programs aiming to increase LLIN use should focus on improving knowledge and attitudes.
- *Age groups*: the variable was retained in the model because its *p*-value was less than 20% in univariate regression. When adjusting the variable for knowledge and attitudes, the effect remained insignificant with a *p*-value = 0.3, suggesting that age alone may not be a key determinant of net use in our context. This means that age alone may not be a key determinant of LLIN use in this context and that behavioral factors are more likely to influence. This result differs from the findings in Nigeria, where younger women (under 30 years of age) were less likely to use LLINs than older women (11). The difference may be the result of contextual differences in health education, or cultural norms influencing preventive behavior among women in child bearing age. In this situation, programs should target all age groups to increase the LLIN use.
- *Are you currently pregnant*: the fact that OR is equal to 1.46 with 95% CI = 1.05–2.05 in the final model means that pregnant women tend to use LLINs more than non-pregnant women, adjusting for other variables in the model. This suggests that women may adopt protective behaviors more consistently during pregnancy. This result is consistent with that in Nigeria, where pregnant women who used LLINs were more numerous in their study (11). This result highlights the importance of antenatal care visits to reinforce LLINs use among vulnerable population. Strengthening targeted interventions for pregnant women remain important, but ensuring sustained LLIN use beyond pregnancy is equally critical to maximize protection.
- *Knowledge about malaria*: A knowledge variable was maintained in the model. This refers to the knowledge that “malaria is caused by mosquito bites”. Such evidence underscores the critical role of basic disease awareness in shaping preventive behaviors. Accurate knowledge may increase risk perception and motivate protective behavior. In Ghana mothers with good knowledge of malaria were 12 times more likely to use mosquito nets for their children compared to those with poor knowledge (22). Strengthening community knowledge may enhance LLIN use and contribute to malaria efforts.
- *Attitudes toward malaria and LLINs*: these attitude variables were significantly associated with LLIN use by women of childbearing age with five dimensions introduced into the model, found to be statistically associated with LLINs use, with *p*-values below 0.001. These results highlight that changing attitudes is an essential lever for improving LLINs adoption. Indeed, the reluctance observed seems to be more related to negative perceptions of malaria and LLINs than to a lack of access or material resources. Interventions should prioritize shaping favorable attitudes through communication, education and community engagement, as these dimensions appear central to sustaining high LLIN utilization.

This study has some very important limitations that should be highlighted:

- The cross-sectional nature of the data does not allow us to establish a causal relationship between the factors identified and the use of LLINs;
- The self-reported nature of the data exposes the results to social desirability bias, particularly for health-related behaviors.

Nevertheless, the study has a number of advantages, including:

- The data is recent, allowing for an update on behaviors related to the use of LLINs;
- It covers four out of eight regions of Guinea;
- It takes into account variables related to knowledge and attitudes toward malaria.

That is why it would be important to consider conducting studies on the reasons why women of childbearing age in these same regions do not use LLINs, as well as an assessment of the impact of awareness messages on these women, which would help refine interventions and strengthen other available means of prevention. The focus on women of childbearing age is a strategic choice, in line with public health priorities and malaria control programs. Future work should be beyond descriptive associations to include longitudinal, qualitative, intervention and comparative studies, linking findings to policy and strategies for malaria control.

## 5 Conclusion

This study highlights the main factors associated with the use of LLINs by women of childbearing age in four regions of Guinea. The percentage of LLIN use by women on the day before the survey was relatively high, at 64.2%.

The results suggest that communication strategies should now focus on transforming women’s knowledge and attitudes by mobilizing more engaging, participatory approaches that are adapted to local realities. In light of the above, health authorities must strengthen communication on the protective role of LLINs, emphasizing their effectiveness and importance in malaria prevention. In addition, they must develop campaigns focused on changing attitudes, using positive messages, local testimonials, and community-based approaches involving women. As for women of childbearing age, they must maintain and reinforce the regular use of LLINs, without neglecting all other malaria prevention measures, including environmental sanitation and early access to care.

## Data Availability

The data analyzed in this study is subject to the following licenses/restrictions: The data are not publicly available and are at the disposal of the Ministry of Health of Guinea. Requests to access these datasets should be directed to Almamy Amara Toure through alamine82@outlook.fr.
