# Supplementary material for: Factors associated with the use of long-lasting insecticide-treated mosquito nets among women of childbearing age: secondary analysis of data from a KAP survey in four regions of Guinea 2023
Source: Front Public Health. 2026 Apr 8;14:1770818. doi: 10.3389/fpubh.2026.1770818 (PMC13101759; doi:10.3389/fpubh.2026.1770818)
Supplement: Supplementary file 1 [file Table_1.docx]

**Supplementary material (Model diagnostics and selection)**

In Model 2, the collinearity assessment yielded generalized variance inflation factors (VIF) below 1.2, which is well below commonly used thresholds and supports the robustness of the model with respect to independence among explanatory variables. A stepwise selection procedure based on the Akaike Information Criterion (AIC) excluded marital status, age groups, and the knowledge item “Does sleeping under an LLIN prevent malaria?” because their inclusion did not meaningfully improve the predictive quality of the model. In addition, the Hosmer–Lemeshow test did not show any significant difference between observed and model-predicted probabilities (p > 0.05), indicating good model fit and suggesting that the logistic regression predictions are consistent with empirical observations. The model can therefore be considered well-calibrated for estimating the probability of LLIN use among women of childbearing age in the four regions of Guinea.
